# Mice Lacking the Intestinal and Renal Neutral Amino Acid Transporter SLC6A19 Demonstrate the Relationship between Dietary Protein Intake and Amino Acid Malabsorption

**DOI:** 10.3390/nu11092024

**Published:** 2019-08-29

**Authors:** Kiran Javed, Stefan Bröer

**Affiliations:** Research School of Biology, The Australian National University, Canberra, ACT 2600, Australia

**Keywords:** amino acid transporter, metabolic health, phenylketonuria, protein restriction

## Abstract

Dietary protein restriction has beneficial impacts on metabolic health. B^0^AT1 (SLC6A19) is the major transporter of neutral amino acids at the intestinal epithelia and absorbs the bulk of the diet-derived neutral amino acids from the intestinal lumen. It also reabsorbs neutral amino acids in the renal proximal tubules. Mice lacking B^0^AT1 show cellular outcomes of protein restriction, such as high FGF21 levels and low mTORC1 activity. Moreover, they have improved glucose homeostasis and resist diet-induced obesity. In this study, we investigated the relationship between protein restriction and dietary protein intake in C57Bl6/J wild-type (*wt*) and SLC6A19-knockout (SLC6A19*ko*) mice. When SLC6A19*ko* mice were fed diets containing 5%, 25%, or 52% of their total calories derived from protein, no differences in food intake or weight gain were observed. All essential amino acids significantly positively correlated with increasing dietary casein content in the *wt* mice. The SLC6A19*ko* mice showed reduced postprandial levels of essential amino acids in plasma, particularly following high-protein diets. Upon fasting, essential amino acids were the same in the *wt* and SLC6A19*ko* mice due to reduced amino acid catabolism. Bacterial metabolites originating from amino acid fermentation correlated with the dietary protein content, but showed a complex profile in the blood of the SLC6A19*ko* mice. This study highlights the potential of SLC6A19 as a knock-out or inhibition target to induce protein restriction for the treatment of metabolic disorders.

## 1. Introduction

Restriction of dietary protein or of specific amino acids has been proposed as a potential treatment for metabolic syndrome [1,2]. Chronic protein restriction has long been associated with an array of beneficial metabolic responses, such as enhanced insulin sensitivity, reduced body weight, increased energy expenditure, and longevity [3,4,5,6,7,8]. The metabolic hormone FGF21 is important for the mediation of beneficial metabolic responses to protein restriction [9,10,11,12,13,14], but the extent of its contribution is still unknown [15]. Although key amino acids responsible for the maintenance of metabolic health remain unidentified, neutral amino acids are apparently critical for this function [16,17]. Restriction of branched-chain amino acids (BCAAs; leucine, isoleucine, valine), individually or in combination, is thought to improve metabolic health due to their effects on signaling via insulin, insulin-like growth factor 1 (IGF1), and mammalian target of rapamycin (mTOR) [1,18]. Reduction of dietary BCAAs reduces fat mass [19] and improves muscular insulin sensitivity in rodents [2]. Methionine or tryptophan restriction has been strongly associated with longevity [20,21], and methionine restriction alone has been shown to reduce body weight and fat mass and to improve glucose metabolism [19,21,22]. This phenotype has been attributed, at least partially, to FGF21 upregulation [23], which in turn increases energy expenditure by promoting browning of the white adipose tissue (WAT) [12,24]. Although methionine and leucine restriction drive some common mechanisms related to protein restriction that underlie improved metabolism, the metabolic effects of the former are more potent than those of the latter [25]. Recently, threonine and tryptophan have emerged as key mediators of protein restriction [3]. The link between FGF21 and dietary protein restriction was first made by Laegar et al. [9,10], renewing interest in its potential for treating metabolic disorders, such as diabetes and fatty liver disease [12,26].

While the benefits of protein restriction are well-documented, its usefulness as a dietary intervention is largely restricted to carefully controlled animal experiments. With the possible exception of the vegan diet [27], selectively reducing the intake of individual amino acids to a beneficial level is difficult in human nutrition. However, many physiological outcomes of protein restriction are replicated in SLC6A19-knockout (SLC6A19*ko*) mice, for example, increased FGF21 levels, reduced mTORC1 signaling in liver, intestine, and adipose and muscle tissues, and insulin-independent glucose removal [28,29]. SLC6A19 is the major transporter of neutral amino acids at the apical side of small intestine epithelia and renal proximal tubular epithelia, functioning as the major mediator that delivers neutral amino acids to the systemic circulation [30,31]. Consistent with the metabolic effects of protein restriction, SLC6A19*ko* mice have reduced body weight, improved glucose tolerance, reduced plasma and liver fatty acids, and browning of subcutaneous white adipose tissue when compared to wild-type (*wt*) mice [28]. The potential use of SLC6A19 as a target to improve metabolic disease is further exemplified by the ability of SLC6A19*ko* to normalize the elevated levels of phenylalanine in a mouse model of phenylketonuria [32]. Lack of human SLC6A19 results in Hartnup disorder [31,33], a largely asymptomatic protein-malabsorption syndrome characterized by high levels of neutral amino acids in the urine. This phenotype is fully replicated in SLC6A19*ko* mice, showing elevated levels of neutral amino acids in urine and faeces due to lack of renal and intestinal transporters, respectively [34]. Beneficial effects of protein restriction, such as upregulation of FGF21 and improved glucose tolerance, as observed in the SLC6A19*ko* model, are yet to be confirmed in Hartnup patients. We propose that pharmacological blockage of SLC6A19 [35,36] can achieve replication of beneficial effects of protein restriction without the need to implement strict dietary habits that are practically difficult to maintain. Inhibition of SLC6A19 may have further benefits, as it causes amino acids to move further distal in the intestine, where it triggers the release of incretins, namely, glucagon-like peptide-1 (GLP-1) and glucose-dependent insulinotropic peptide (GIP) [28]. The presence of amino acids in the distal intestine can be readily detected by the appearance of bacterial amino acid fermentation products [34].

We therefore investigated the role of SLC6A19 under different protein diets using an SLC6A19*ko* mouse model as a surrogate for its complete pharmacological inhibition. Several studies have previously used untargeted metabolomics to predict dietary outcomes by studying different biological fluids, such as urine or plasma [37,38]. Here, we aimed to investigate the nexus between dietary protein composition and intestinal protein absorption.

## 2. Materials and Methods

### 2.1. Mice and Diets

Mouse experiments were approved by the animal experimentation ethics committee of the Australian National University (protocol: A2016/41). Both male and female C57Bl6/J and SLC6A19*ko* (with the same background) mice were obtained from the Australian Phenomics Facility of Australian National University and were used between six and eight weeks of age. Mice had free access to water and chow unless restricted during experiments. Specialized diets were purchased from Specialty Feeds (Perth, Western Australia, Australia); the manufacturer ensured to provide chow with the same total calorie content (isocaloric) but with different protein-to-carbohydrate ratios and with constant lipid content (Supplementary Table S1). Casein was used as the primary protein source, which was balanced with starch as the main carbohydrate source. The diets contained all essential components, such as soybean oil, vitamins, minerals, and amino acids, based on the laboratory mouse diet AIN-93G (Specialty Feeds, Perth, Western Australia, Australia). In the first experiment, 30 C57Bl6/J (*wt*) mice were randomly assigned to isocaloric diets with casein contents of 5% (low protein or LP), 25% (standard protein or SP) and 52% (high protein or HP) protein (*n* = 10 *wt* per group). The diets continued for 14 days before collection of plasma, urine, and faecal samples. To synchronize the nutritional states of the mice, they were fasted for 6 h and were then allowed to resume feeding their specified diet for 1 h before being culled by cervical dislocation. In the second experiment, five SLC6A19*ko* and *wt* mice were fed consecutively 5%, 25%, and 50% casein diets. Blood samples were collected in the fasting and refed states, consistent with previous studies investigating the metabolic effects of SLC6A19 deficiency mainly after nutrient intake [34]. The plasma samples were isolated in potassium ethylenediaminetetraacetic acid (kEDTA) tubes (Sarstedt, Nümbrecht, Germany) and stored at −80 °C immediately. All samples were collected from 3:00 to 6:00 pm and the feeding patterns were documented.

### 2.2. Untargeted Metabolomics of Urine, Faecal, and Plasma Samples

All samples were processed using a previously established metabolomics workflow [34]. Briefly, urine (after pretreatment with urease), faeces, and plasma samples were extracted in a mixture of methanol and water (9:1, *v*/*v*), with ribitol (100 ng/μL, Sigma-Aldrich, St. Louis, MO, USA) added as the internal standard. Samples were then mixed by a vortex at 2500 rpm for 10 min using a multi-plate shaker (Biosan, Riga, Latvia). After centrifugation at 13,400× *g* for 10 min, the supernate was transferred to a new tube and lyophilized. A GERSTEL MPS2 multipurpose sampler (GERSTEL GmbH & Co. KG, Mülheim an der Ruhr, Germany) was used to derive and inject the samples under a splitless mode into a gas chromatograph (GC; Agilent 7890A, Agilent Technologies, Palo Alto, CA, USA), coupled with a single-quadrupole mass spectrometer (Agilent 5975C, Agilent Technologies, Palo Alto, CA, USA). The GC was equipped with a 10 m EZ-Guard column and a J&W VF-5 MS column (30 m × 0.25 mm × 0.25 μm; Agilent Technologies, Palo Alto, CA, USA). The injector was set to 230 °C, and helium was used as a carrier gas at 1 mL/min. The oven was held initially at 70 °C for 1 min; this was subsequently increased at 15 °C/min to reach 325 °C, which was held for 3 min, making up a 21 min run duration. The electron impact (EI) ion source and quadrupole were kept at 250 °C and 150 °C, respectively. The filament current was set at 70 eV. The auxiliary transfer line was kept at 260 °C, and the quadrupole mass analyzer was operated in full MS scan acquisition mode from 40–600 m/z using a scan rate of 3.6 Hz. The solvent delay was 5.60 min. A quality control (QC) sample (all samples pooled) was injected several times at the start of the run and after every eighth sample. Blank samples were run at the start and end of the batch, and all samples were run randomly. Agilent Mass Selective Detector (MSD) Mass hunter software (version E.02) was used for data acquisition. The data were then converted into the .abf format to import into MS-DIAL [39]. Data were processed and identified using standard settings of the software. The GOLM library was imported into the software to identify peaks with <3 retention index (RI) difference and a mass spectrum similarity score of >850 [40]. The search for further annotation of the peaks was broadened by using public gas chromatography mass spectrometry (GC-MS) libraries that came with MS-DIAL. Ribitol (the internal standard) was used to check the reproducibility of its peak height and retention time in each tested sample. The data were filtered as previously reported [34]. The peak height was used as a measure of relative quantification of metabolites using the quantifier ion characteristic of a corresponding metabolite. Metabolomics dataset is presented in the form of a metadata (Supplementary Table S4).

### 2.3. Statistical Analyses

Principal component analysis (PCA) was performed using MetaboAnalyst 4.0 [41]. PCA was used to visualize the clustering difference between metabolic profiles of all sample groups. Data were then subjected to volcano plot analysis in Graphpad Prism Version 7 (GraphPad Software, La Jolla, CA, USA) to calculate fold-changes between LP vs. SP and HP vs. SP diets using the *p*-value <0.001 [42] as a threshold for significance. After log-transforming the data, all metabolites with a fold-change (FC) <0.8 for LP/SP and ≥1 for HP/SP and a *p*-value of <0.001 were selected for further analysis. The line plots of metabolites that passed QC were generated using GraphPad Prism Version 5.01 (GraphPad software, La Jolla, CA, USA). The statistical significance of differences between individual metabolites was calculated by using ANOVA, with *p*-values representing the difference of within-group variance and between-group variance.

## 3. Results

### 3.1. Plasma Metabolome Reflects Changes in Dietary Protein Intake

**Figure 1:**
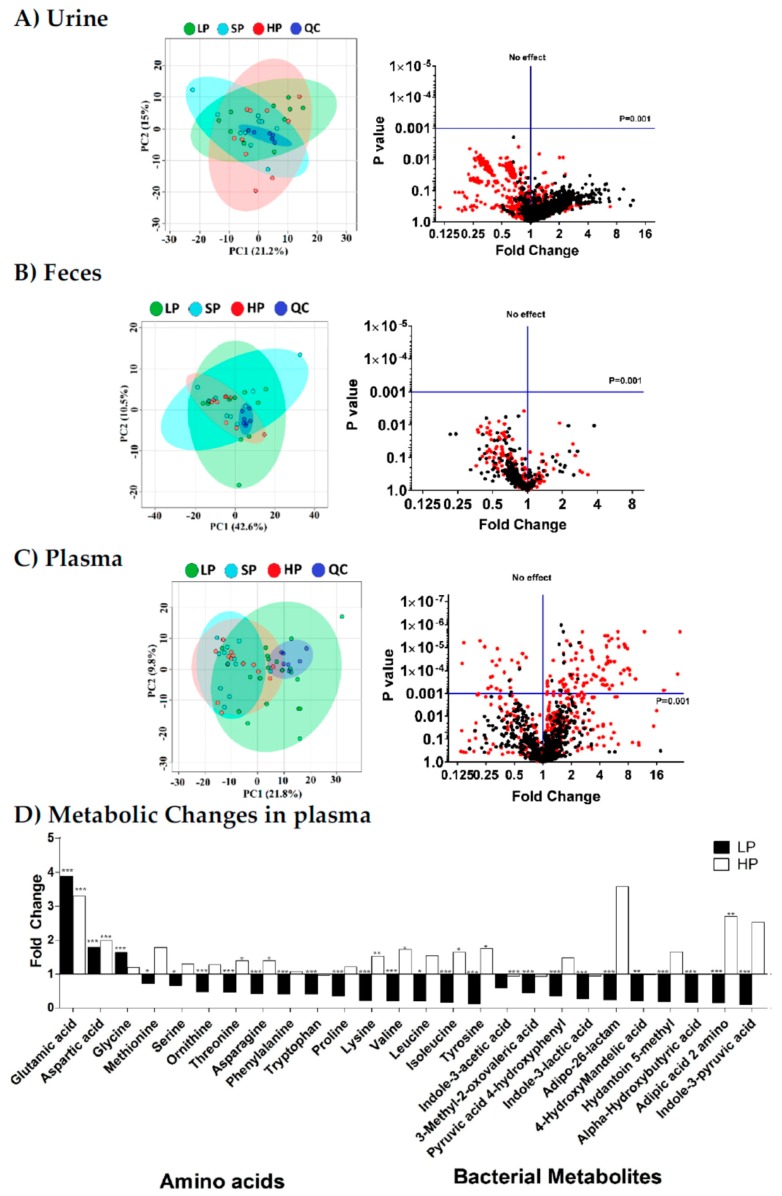
Metabolomic profiles of (**A**) urine, (**B**) faeces, and (**C**) plasma samples of *wt* mice on diets with low (LP—green), standard (SP—light blue), or high (HP—red) protein content (panels on the left). To synchronize the nutritional states of the mice, plasma samples were collected in the re-fed state, i.e., mice held for 14 days on the specified diet were fasted for 6 h and then re-fed with the same diet for 1 h before sample collection. Using standard settings, principal component analysis (PCA) could not discriminate between the three conditions. The quality control (QC) (dark blue) samples appear as tight clusters. The panels on the right side show the volcano plots constructed from all mass features in which the x-axis represents fold-change and the y-axis represents significance (*p*-value). The plot shows the log-converted fold-change of metabolites between the LP and SP groups as red dots and between the HP and SP groups as black dots in (**A**) urine, (**B**) faeces, and (**C**) plasma samples of *wt* mice. The horizontal blue line represents the statistical significance cutoff, and the vertical blue line represents no change. (**D**) Amino acid and bacterial metabolite changes in plasma. Fold-changes in LP (black) and HP (white) groups are compared to the SP group. Statistical significance is presented as follows: * for *p*-values between 0.05 and 0.01; ** for p-values between 0.01 and 0.001; *** for *p*-values <0.001 (*n* = 10 per group).

High-protein or low-protein diets are frequently used experimentally, but how these diets affect metabolite homeostasis is unknown. In this study, we used untargeted metabolomics to identify the metabolites that correlate with dietary protein (casein) content and compared the metabolic profiles of the diets between SLC6A19*ko* and *wt* mice. Mice were fed isocaloric diets with different amounts of casein (5% (LP), 25% (SP), and 52% (HP) *n* = 10 per group) for 14 days. The average body weights of *wt* mice assigned to the LP, SP, and HP groups were 22.5 ± 3.3, 23.4 ± 4.8, and 23.6 ± 3.8 g ($\bar{x}\pm SD$), respectively, and did not change significantly during the dietary intervention. Despite the large variation of starch vs. casein, PCA of global metabolite profiles in plasma, urine, and faecal samples collected after the dietary intervention failed to clearly distinguish between the diets of different protein contents in the refed state (14 days of feeding/6 h fasting/1 h feeding) (Figure 1; left panels). These results highlight two important physiological concepts. (1) Protein-derived amino acids are efficiently absorbed by the intestines of the *wt* mice. Even on the HP diet, mice did not show increased faecal amino acid content. (2) Metabolite homeostasis in plasma is tightly regulated. Except for certain amino acids (see below) and their bacterial metabolites, metabolite concentrations changed less than two-fold when the casein content of the diet was altered. Because PCA showed some clustering of plasma samples of LP mice away from SP or HP mice, we used the volcano plot analysis to identify metabolites that changed significantly in the plasma of *wt* after changing the dietary protein content (Figure 1; right panels). In the plots, fold-changes (FC) of metabolites between the LP and SP groups are shown as red dots, whereas the fold-changes between HP and SP are shown as black dots. Of the three sample types, only plasma samples showed significant metabolite changes with a *p*-value <0.001. We chose this *p*-value to focus on reliable indicators of dietary protein content [42]. Only essential amino acids and bacterial amino acid-derived metabolites in plasma samples reached this *p*-value between the LP, SP, and HP groups. The abundance of most essential amino acids, including BCAAs, threonine, tryptophan, tyrosine, and phenylalanine, correlated with the dietary protein content. Aspartic acid (FC LP/SP: 1.8 and FC HP/SP: 2.0) and glutamic acid (FC for LP/SP: 3.9 and FC for HP/SP: 3.3) showed increased abundance in both LP and HP groups. The metabolites presented in Figure 1D positively correlated with the dietary casein content and are, therefore, useful biomarkers of protein restriction in animal models, such as SLC6A19*ko* mice.

### 3.2. Impact of Dietary Protein on Food Intake and Body Weight of SLC6A19ko and wt Mice

**Figure 2:**
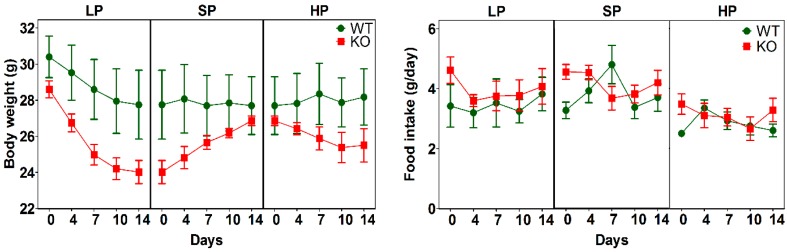
Body weight and food intake during diet intervention. Both parameters were recorded for male SLC6A19*ko* and *wt* mice on low protein (LP = 5% protein energy), standard protein (SP = 25% protein energy), and high protein (HP = 52% protein energy) diets. Data are shown as mean ± SEM (*n* = 5 male mice per group).

Next, we compared the metabolic profiles of SLC6A19*ko* and *wt* mice by placing five male mice from each group consecutively on LP, SP, and HP diets. Blood samples were taken after 14 days of each protein diet followed by fasting for 6 h (fasting sample) and re-feeding the same diet for 1 h (re-fed sample). As reported previously, SLC6A19*ko* mice had slightly lower body weights due to slower growth after weaning [29] and showed a more pronounced weight loss on the LP diet (Figure 2) compared to *wt* mice. This weight loss on the LP diet was most likely caused by the use of alternative fuels to maintain energy demands [28]. Body weight returned to normal when the mice were switched back from the LP to the SP diet. No significant differences were found in food intake between animal groups during the 14-day dietary intervention. However, both *wt* and SLCA19*ko* mice tended to reduce their food intake when on the HP diet.

### 3.3. Amino Acid Levels in the Re-Fed State

**Figure 3:**
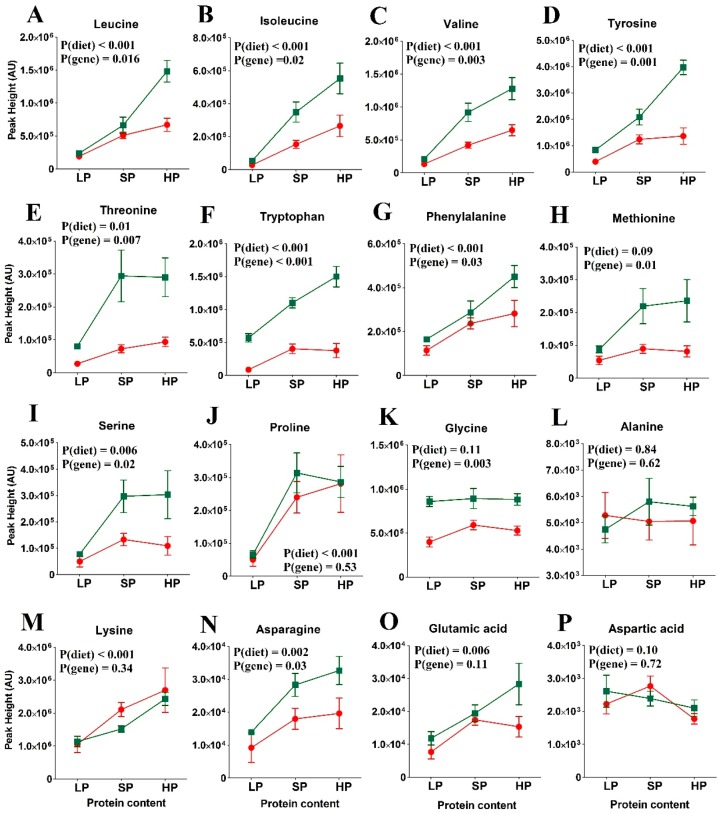
Postprandial amino acid abundance in SLC6A19*ko* (red) and *wt* (green) mice on diets with low (LP), standard (SP), or high protein (HP) contents (*n =* 5 per group). Amino acids: (A) Leucine, (B), Isoleucine, (C) Valine, (D) Tyrosine, (E) Threonine, (F) Tryptophan, (G) Phenylalanine, (H) Methionine, (I) Serine, (J) Proline, (K) Glycine, (L) Alanine, (M) Lysine, (N) Asparagine, (O) Glutamic acid and (P) Aspartic acid. Levels of all essential amino acids positively correlated with the dietary protein content in the *wt* mice. SLC6A19*ko* mice exhibited reduced levels of neutral amino acids due to their dependence on SLC6A19 for amino acid absorption. Note the most prominent difference in plasma amino acid levels between SLC6A19*ko* and *wt* was observed following the HP diet. Non-essential amino acids showed no difference between SLC6A19*ko* and *wt* mice. The data points represent $\bar{x}\pm SEM$ (*n* = 5 per group). *p*-values were calculated by ANOVA comparing within-group and between-group variance of the diet effects (diet) and genotype effects (gene).

Figure 3 and Supplementary Table S2 shows the abundance of selected plasma amino acids in the re-fed state. In the *wt* mice (green symbols), peaks of essential amino acids clearly correlated with dietary protein content. Some non-essential amino acids (glycine, alanine, aspartic acid) remained constant, while proline, serine, asparagine, and glutamic acid followed the trend of the essential amino acids. While the same trend was observed in SLC6A19*ko* mice, it was not significant. The most pronounced difference between the *wt* and SLC6A19*ko* mice was observed for plasma levels of threonine and tryptophan on all the tested protein diets. With the exception of the alanine and proline levels, the postprandial levels of neutral amino acids were significantly reduced in SLC6A19*ko* mice after an HP meal, indicating protein malabsorption in SLC6A19*ko* mice as opposed to *wt* mice. Postprandial plasma levels of valine, isoleucine, and leucine strongly correlated with dietary protein content; this correlation was blunted in SLC6A19*ko* mice. Overall, the genotype effect on essential amino acids was more pronounced in the HP group than the LP or SP groups. The data from the SLC6A19*ko* mice also showed that the SP and HP diets resulted in amino acid levels that were comparable or higher than those in the *wt* LP group (Figure 3). Thus, elevated consumption of protein compensated for SLC6A19 deficiency.

**Figure 4:**
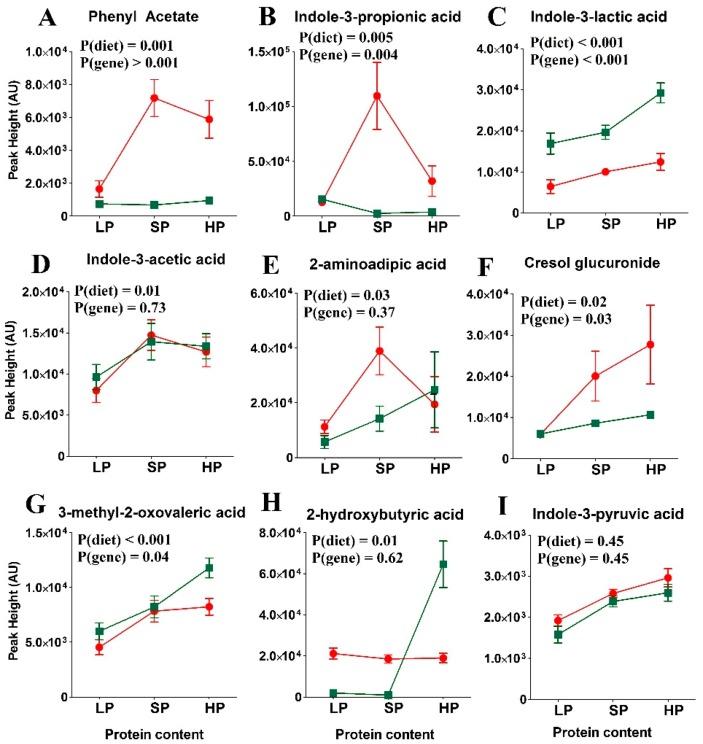
Abundance of amino acid-derived metabolites in SLC6A19*ko* (red) and *wt* (green) mice in the postprandial state on diets with low (LP), standard (SP), or high protein (HP) contents. Amino acid-derived metabolites: (A) Phenyl acetate, (B) Indole-3-propionic acid, (C) Indole-3-lactic acid, (D) Indole-3-acetic acid, (E) 2-aminoadipic acid, (F) Cresol glucuronide, (G) 3-methyl-2-oxovaleric acid, (H) 2-hydroxybutyric acid and (I) Indole-3-pyruvic acid. Amino acid-derived bacterial metabolites showed an increase in abundance in SLC6A19*ko* compared to *wt* mice. The data points represent $\bar{x}\pm SEM$ (*n* = 5 per group). *p*-values were calculated by ANOVA comparing within-group and between-group variance of the diet effects (diet) and genotype effects (gene).

In the previous experiment, we found that a few bacterial metabolites also varied with protein abundance in the postprandial state. Next, we measured the abundance of these bacterial metabolites with respect to the genotype and diet in more detail. Phenyl acetate, cresol glucuronide, and indole-3-propionic acid distinguished between *wt* and SLC6A19*ko* mice, consistent with previous results [34] (Figure 4 and Supplementary Table S2). Their elevated levels can be attributed to both high dietary protein content and greater bacterial fermentation of amino acids in SLC6A19*ko* mice due to increased availability of the nutrients in the distal intestine. In the LP group, bacterial metabolites were hardly detectable regardless of the genotype, indicating complete absorption of amino acids and peptides before they could be fermented by the bulk of the microflora in the distal intestine.

### 3.4. Amino Acid Levels in SLC6A19ko and wt Mice Following the Fasting State

**Figure 5:**
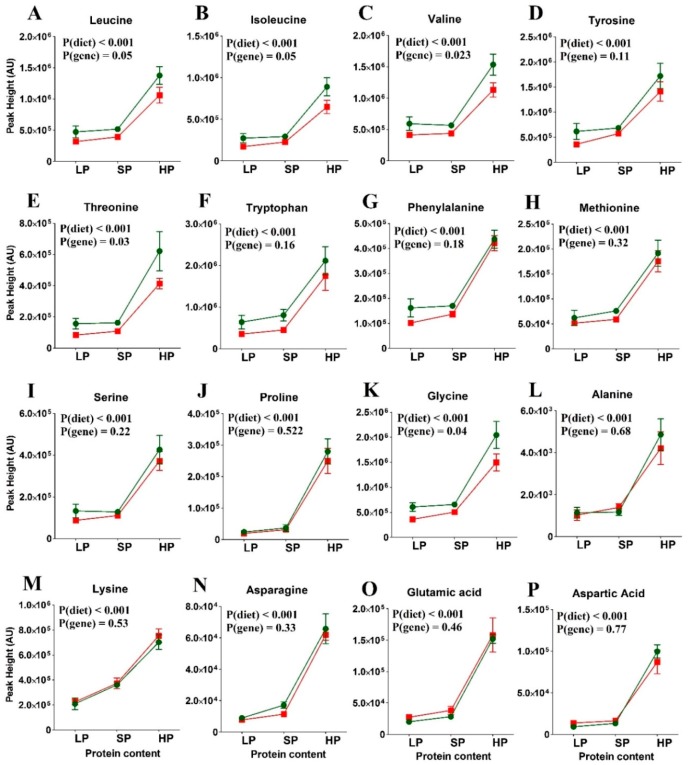
Plasma amino acid levels in SLC6A19*ko* (red) and *wt* (green) mice on diets with low (LP), standard (SP), and high protein (HP) contents in the fasted state. Amino acids: (A) Leucine, (B), Isoleucine, (C) Valine, (D) Tyrosine, (E) Threonine, (F) Tryptophan, (G) Phenylalanine, (H) Methionine, (I) Serine, (J) Proline, (K) Glycine, (L) Alanine, (M) Lysine, (N) Asparagine, (O) Glutamic acid and (P) Aspartic acid. The error bars represent $\bar{x}\pm SEM$ (*n* = 5 per group). *p*-values were calculated by ANOVA comparing within-group and between-group variance of the diet effects (diet) and genotype effects (gene).

Levels of amino acid metabolites and bacterial metabolites in SLC6A19*ko* and *wt* mice were also assessed after fasting (Figure 5 and Supplementary Table S3). In contrast to the postprandial increase in plasma amino acid levels between the LP and SP diets, plasma levels remained largely constant between the two diets following the fasting state. However, all amino acids showed a significant fold-change >1.2 when mice were switched to the HP diet (52%). Consistent with previous observations [34], no significant differences in plasma amino acid levels were observed between *wt* and SLC6A19*ko* mice on the SP diet; this observation was also true for the LP diet. On the HP diet, the abundance of all amino acids increased in SLC6A19*ko* mice, similarly to *wt* mice, but small differences were observed for isoleucine, valine, and threonine.

**Figure 6:**
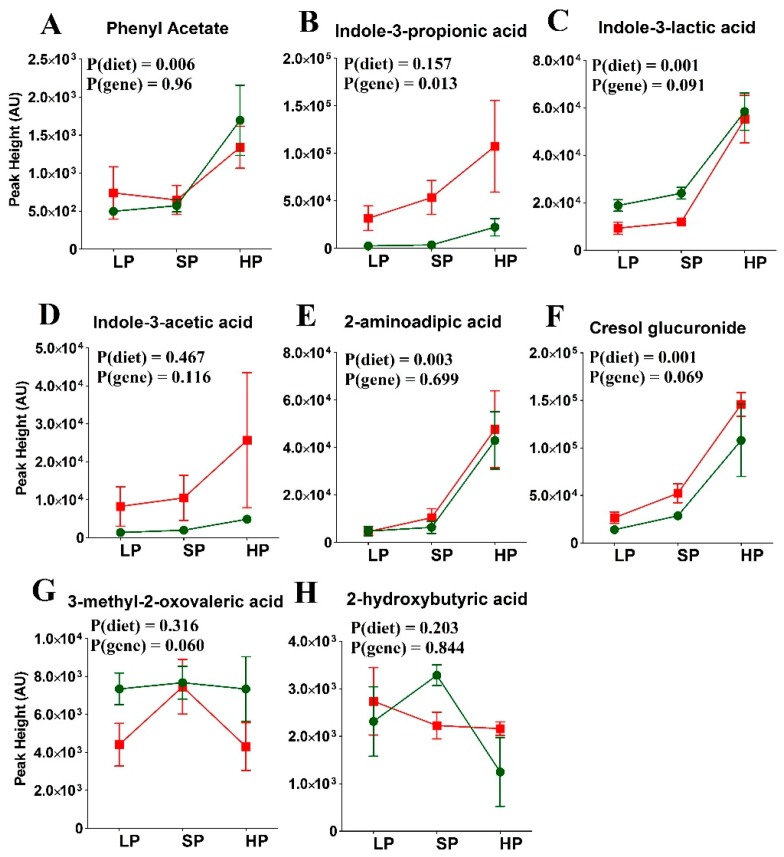
Abundance of amino acid-derived metabolites in the plasma of SLC6A19*ko* (red) and *wt* (green) mice on diets with low (LP), standard (SP), or high protein (HP) contents in the fasted state (*n =* 5). Amino acid-derived metabolites: (A) Phenyl acetate, (B) Indole-3-propionic acid, (C) Indole-3-lactic acid, (D) Indole-3-acetic acid, (E) 2-aminoadipic acid, (F) Cresol glucuronide, (G) 3-methyl-2-oxovaleric acid and (H) 2-hydroxybutyric acid. No significant differences (*p*-value of <0.05) were observed between the genotypes. The error bars represent $\bar{x}\pm SEM$ (*n* = 5 per group). *p*-values were calculated by ANOVA comparing within-group and between-group variances of the diet effects (diet) and genotype effects (gene).

Bacterial amino acid-derived metabolites also showed similar levels in *wt* and SLC6A19*ko* mice following fasting (Figure 6 and and Supplementary Table S3). Indole-3-propionic acid and indole-3-acetic acid levels were higher in the SLC6A19*ko* than in *wt* mice, but were not considered significant due to relatively large standard deviations of the HP samples. Bacterial amino acid metabolites derived from aromatic and cationic amino acids increased, particularly when the mice were on an HP diet. Metabolites derived from BCAAs did not change with dietary protein content.

Overall, essential amino acids and bacterial amino acid-derived metabolites were reliable indicators of the dietary protein composition when measured in the postprandial state.

## 4. Discussion

Restriction of dietary protein is emerging as a nutritional concept that improves metabolic health in mice; however, with the possible exception of a vegan diet [27], it cannot be easily applied to dietary recommendations in humans. We previously showed that ablation of SLC6A19 generated a metabolic phenotype similar to that achieved by dietary protein restriction [28,32]. BCAAs, namely, methionine and threonine, were investigated as candidate amino acids, representing the main drivers of improved metabolic health [7,43,44]. Notably, these amino acids are all substrates of SLC6A19 [30]. In this study, we aimed to investigate the nexus between protein malabsorption and dietary protein content. We previously identified groups of metabolites that can serve as biomarkers of protein restriction and malabsorption, mostly amino acids and amino acid-derived products of bacterial fermentation [34]. Experimentally, these biomarkers are best determined 1 h after feeding, which in mice can be synchronized by a short fasting period (6 h), followed by offering chow. In our hands, this is a reliable method to induce feeding before sample collection. Under these conditions, the peak height of essential amino acids and that of bacterial amino acid-derived metabolites largely reflect their postprandial absorption on top of low, basal fasting metabolite levels. This is consistent with amino acid absorption time courses in human control subjects and individuals with Hartnup disorder [45]. Two aspects are discussed here: Firstly, the difference between *wt* mice and SLC6A19*ko* mice, and secondly, general trends associated with diets of different protein contents.

Genotype Effects: Due to its location, SLC6A19 plays a pivotal role in modulating the transfer of neutral amino acids from the lumen of the intestine into the blood and further to organs [46,47]. Ablation of SLC6A19 reduces the absorption of all neutral amino acids, but threonine and tryptophan appear to be affected more than other amino acids, while remaining low in the absence of SLC6A19 on the tested diets with different protein contents. Whether these two amino acids underlie the physiological outcomes in SLC6A19*ko* mice remains to be proven [28]. The absorption of other neutral amino acids, such as BCAAs, phenylalanine, and methionine, was slightly reduced on an SP diet, but this outcome was prominent when dietary protein content was increased to 52%. Fasting amino acid levels were not affected by the genotype. We previously showed that amino acid levels were maintained by reducing amino acid metabolism, as evidenced by lower levels of urea [28], thereby compensating for reduced absorption and increased excretion of amino acids [34].

In addition to neutral amino acid uptake mediated by SLC6A19, neutral amino acids are also absorbed as dipeptides or tripeptides via the peptide transporter PEPT1 (SLC15A1) from the intestine [33,48]. Involvement of SLC15A1 in intestinal amino acid absorption only becomes evident in the presence of high protein loads, while no significant differences in plasma amino acid levels are observed with diets of low or normal protein content [49,50]. A few amino acids, such as valine, isoleucine, proline, and threonine, showed reduced plasma levels in the SLC15A1*ko* mice compared to *wt* mice after administration of a protein-enriched bolus [50].

Our study demonstrated that a lack of SLC6A19 affected the absorption of threonine and tryptophan more than that of other amino acids. This was surprising, as neither amino acid was the preferred substrate of this transporter [35], which may point to redundancy for BCAAs and other large neutral amino acids. The apical amino cationic amino acid transporter b^0,+^AT could mediate the transport of BCAA, but only in exchange with cationic amino acids [51]. However, the existence of an additional neutral amino acid transporter in the apical membrane of the small intestinal epithelia was not proven [52]. There were no changes to the expression of basolateral neutral amino acid transporters, such as LAT2 or TAT1, which could affect tryptophan or threonine absorption (unpublished data). In the large intestine, expression of amino acid transporters ATB^0,+^ and ASCT2 is prominent [52,53,54], and some amino acids may move distally enough in SLC6A19*ko* mice [34,55] to be taken up by these transporters. Threonine is a poor substrate of ATB^0,+^, but a good substrate of ASCT2, while tryptophan is a good substrate of ATB^0,+^, but not accepted by ASCT2 [52]. Thus, it appears unlikely that absorption in the distal intestine compensates for lack of B^0^AT1. PEPT1 also does not strongly discriminate between amino acid composition of the dipeptides or tripeptides [56]. Threonine and tryptophan have recently attracted interest as the limiting amino acids underlying hyperphagia induced by low-protein diets [3]. Despite low levels of circulating threonine and tryptophan levels in SLC6A19*ko* mice, we did not observe an increase in food intake, whereas Zapata et al. (2019) [57] and Solon-Biet et al. (2019) [3] reported that an increase in food intake could be corrected by threonine and tryptophan supplementation. Threonine and tryptophan have also been highlighted as key players behind the beneficial effects of protein restriction, at least in part due to FGF21 upregulation [57]. Previously, methionine restriction was linked to the upregulation of FGF21 [23,25,58], whereas contradictory results were obtained upon leucine restriction [25,59]. The restriction of neutral amino acids absorption in SLC6A19*ko* mice is sufficient to induce upregulation of FGF21 comparable to protein-restricted mice [10,28]. Although SLC6A19 effects are more prominent in mice on high-protein diets, the overall amino acid absorption is similar to that of *wt* mice on low-protein diets. This explains why Hartnup disorder is benign on a protein-sufficient diet. Such observations further suggest that pharmacological inhibition of SLC6A19 is likely to generate the metabolic outcomes associated with dietary protein restriction. Elevated levels of circulating BCAAs have been associated with obesity and insulin resistance [60,61,62], although the causal relationship is still unclear [44,63]. Moreover, elevated amino acid levels could reflect high protein intake [3,8].

Effects of Dietary Protein Content: Previously, we identified bacterial metabolites, namely, p-cresol glucuronide and indole-3-propionic acid, originating from tyrosine and tryptophan fermentation, respectively, as SLC6A19*ko* biomarkers in the intestine [34]. Some bacterial metabolites can be used also as indicators of protein content, particularly postprandially. Phenyl-acetate, indole-3-lactic acid, and 2-aminoadipic acid positively correlated with the protein content. Other bacterial amino acid-derived metabolites, such as indole-3-propionic acid and indole-3-actetic acid, increased with LP and SP diets, but returned back to LP diet levels when given an HP diet. This may reflect changes in the microbiome occurring during the 14-day diet adaptation.

We also observed a direct correlation between dietary protein content and the abundance of essential amino acids in *wt* mice postprandially. Previous studies in rats also reported similar correlations of plasma essential amino acids in the postprandial state [17,64], showing a reduction with a 5% casein diet and an increase with diets containing 60% casein, with the exception of threonine [64]. In fasting mice, in contrast, significant increases in amino acids were only observed when they were fed a high-protein diet. This is also in agreement with previous studies, where fasting amino acid levels remained unchanged when rats were fed with a 6% or 24% protein diet, with the exceptions of serine or glycine [65]. Plasma levels of essential amino acids, especially BCAAs, were previously shown to be significantly reduced with low-protein diets [1,4,64] and elevated when high-protein diets were consumed [3,66].

In summary, amino acids are evidently potent bioactive metabolites whose levels are tightly controlled. However, plasma levels of essential amino acids can predict dietary protein intake in the postprandial state. Low or high protein intake has been shown to affect metabolic health by a variety of mechanisms. Our results indicate that absorption of essential amino acids is reduced significantly in the absence of SLC6A19, especially following a high-protein diet. We conclude that pharmacological inhibition of SLC6A19 is one of the very few strategies that could apply the concept of dietary protein restriction to human nutrition.

## Supplementary Materials

The following are available online at https://www.mdpi.com/2072-6643/11/9/2024/s1. Table S1. Composition of the diet used in the study. Table S2. Abundance of metabolites in SLC6A19*ko* and *wt* mice under diets with low or standard protein content in the postprandial state. Table S3. Abundance of metabolites in SLC6A19*ko* and *wt* mice under low or standard protein diets in the fasted state. Table S4. Metadata from the first dietary intervention of *wt* mice with different protein content of the diet.
